# Ultra‐processed food intake and risk of obesity among schoolchildren aged 8–12 years living in Victoria, Australia

**DOI:** 10.1111/ijpo.70030

**Published:** 2025-06-17

**Authors:** Lachlan Clark, Kristy A. Bolton, Kathellen E. Lacy, Karen Lim, Priscila P. Machado, Carley A. Grimes

**Affiliations:** ^1^ School of Exercise and Nutrition Sciences Deakin University Geelong Victoria Australia; ^2^ Institute for Physical Activity and Nutrition, School of Exercise and Nutrition Sciences Deakin University Geelong Victoria Australia

**Keywords:** Australia, childhood obesity, NOVA, ultra‐processed foods

## Abstract

**Introduction:**

Ultra‐processed foods (UPF) are frequently consumed by children, possibly contributing to childhood obesity. It is unknown if UPF consumption among Australian children differentiates by sociodemographic factors.

**Objectives:**

To describe schoolchildren's intake of UPF across sexes, age, geographic location and socioeconomic status (SES). To analyse associations between UPF intake and indicators of obesity.

**Methods:**

UPF consumption of children aged 8–12 years in Victoria (Australia) was examined using 24‐h dietary‐recall data classified by the NOVA system. UPF intake was compared across sociodemographic groups. Regression analysis explored the association between UPF intake and BMI *z*‐score, overweight/obesity and abdominal obesity.

**Results:**

UPF comprised 47.2% of total energy intake (range 23.7%–72.2%), with no significant differences across sex, age group (8–9 vs. 10–12 years), geographic location or SES. Including all children, there were no associations between UPF intake and obesity indicators. In age‐stratified models, among children aged 10–12 years, a 10% increment in the proportion of UPF in the diet (% g/day) was significantly associated with a 0.07 (95% CI 0.01, 0.12) higher body mass index (BMI) *z*‐score and a 19% (odds ratio 1.19, 95% CI 1.07, 1.33) increase in the odds of central obesity. No associations between UPF intake and indicators of obesity were found in the younger 8‐ to 9‐year‐old group.

**Conclusions:**

UPF contributed greatly to the dietary intake of primary schoolchildren. Among older children, higher intake of UPF was associated with higher BMI *z*‐score and central adiposity. Further longitudinal research in Australian pediatric samples to understand UPF impact upon adiposity outcomes across different stages of childhood is needed.

**Abbreviations**
BMI: body mass index
IOTF: International Obesity Taskforce
IRSD: index of relative disadvantage
NHANES: National Health and Nutrition Examination Survey
SES: socioeconomic status
SONIC: Salt and Other Nutrients In Children
UPF: ultra‐processed food(s)
WC: waist circumference
WtHR: waist‐to‐height ratio

## 1 INTRODUCTION

Global rates of children living with overweight and obesity have dramatically increased in recent decades, from 4% (~52 million) of 5‐ to 19‐year‐olds being above a healthy weight in 1975 to 18% (~320 million) in 2016.^1^ The prevalence of overweight and obesity in Australian children (aged 5–14 years) is even greater, at approximately 25% in 2017–2018.^2^ The health‐related consequences of childhood overweight and obesity are broad, and impact the individual in both the short‐ and long‐term, including chronic inflammation, social disadvantage, cardiovascular risk factors and increased risk of non‐communicable diseases, and thus mortality.^3^

Changing eating patterns have been considered a critical aspect of this increased prevalence of obesity,^1^ with the global food supply increasingly characterized by the mass production and promotion of heavily processed, highly palatable and energy‐dense foods.^4^, ^5^ In the NOVA food classification system,^4^ these products are termed ‘ultra‐processed foods’ (UPF) and are gaining attention in peer‐reviewed literature.^5^, ^6^, ^7^, ^8^, ^9^, ^10^ The prevalence of UPF in the diet is rapidly increasing, with Australians purchasing 134 kg of UPF per capita in 2019^7^—and 47%–54% of Australian children and adolescents' daily energy intake coming from UPF.^11^ Reducing consumption of these foods has become the focal point of many national dietary recommendations from Israel,^12^ Malaysia,^13^ and multiple Latin American countries.^14^, ^15^

Evidence highlighting an association between overweight and obesity measures and UPF intake in adults is fairly robust.^9^, ^16^, ^17^, ^18^, ^19^ This includes one randomized controlled trial and multiple systematic reviews, with one meta‐analysis of 140 577 adults across 12 studies reporting a 10% increase in energy intake from UPF was associated with a significant 6% increased risk of obesity.^16^ However, literature investigating this association in children is less consistent.^20^, ^21^, ^22^ To date, four systematic reviews of observational studies have been conducted,^21^, ^23^, ^24^, ^25^ whilst findings generally point towards a positive association between intake of UPF and indicators of overweight and obesity,^24^ there are some inconsistencies across study types, with cross‐sectional studies mostly reporting null associations compared to positive associations within longitudinal studies.^21^, ^23^, ^25^ Overall, research has primarily been conducted in Latin American samples,^24^, ^25^ which does not account for differences in food production technologies or dietary habits across cultures and countries.^21^ Additionally, few studies utilize central adiposity as a marker of obesity.^26^

Sociodemographic disparities in dietary patterns exist^27^; however, few studies have explored the relationship between UPF and socioeconomic status (SES) in children.^8^, ^10^ Along with little data examining the impact of geographic location,^27^ and weak evidence regarding a consistent trend in the impact of sex on UPF consumption.^8^, ^10^ Finally, most research has been conducted in Latin America,^22^, ^28^ the United States,^10^, ^20^ and the United Kingdom,^8^ with no studies examining the relationship among Australian children.

This study aimed to describe the intake of NOVA food groups^4^ (specifically UPF) in primary schoolchildren aged 8–12 years overall, and examine associations between UPF consumption and age, sex, geographic location and SES. Additionally, it aimed to investigate the association between consumption of UPF and indicators of obesity in children. Understanding differences in UPF consumption by sociodemographic variables and its relationship with obesity will inform the development of future approaches to improving the diets of children living in Victoria, Australia.

## 2 METHODS

### 2.1 Data source and population

**FIGURE 1:**
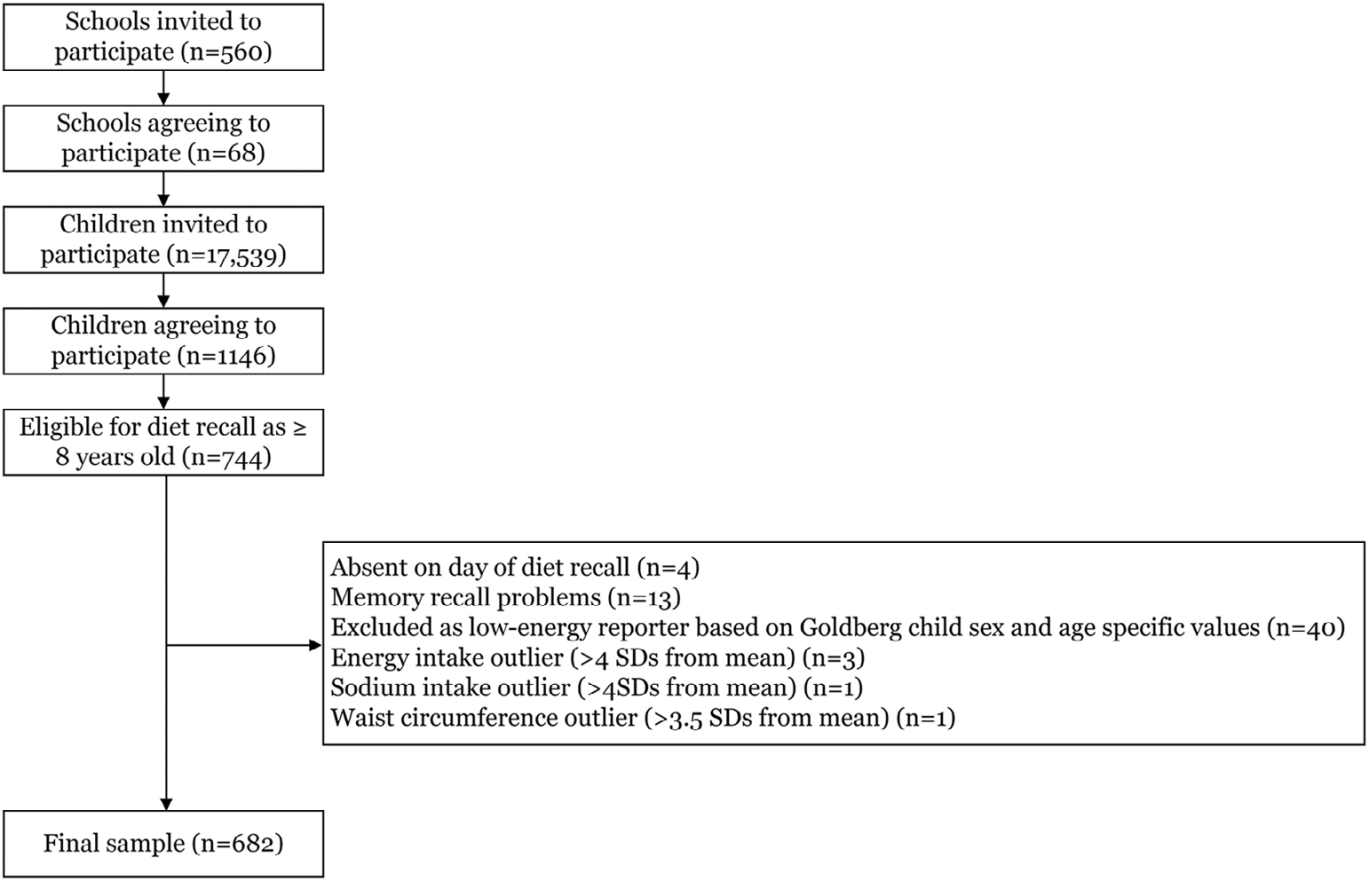
Participant flow chart. SD, standard deviations.

Data for this study comes from the Salt and Other Nutrients In Children (SONIC) Study, which was a cross‐sectional study of primary schoolchildren located in Victoria, Australia conducted in 2010–2013 (Cohort 1) and 2018–2019 (Cohort 2).^29^, ^30^ Further information regarding the study protocol has been reported elsewhere.^30^ Briefly, in 2010–2013, a convenience sample of Victorian primary schools was selected and invited to participate. In 2018–2019, we sought to recruit schools comparable to the previous sample. This was accomplished by firstly inviting the previous sample of schools to participate, and following this inviting additional schools that were comparable with respect to the mix of non‐government and government schools and the spread of schools across levels of socioeconomic disadvantage.^29^ A total of 560 schools were invited to participate, with 68 agreeing to do so (12% response rate). From these schools, 17 539 students were invited to participate in the study, with 1146 agreeing (7% response rate).^29^, ^30^ In this analysis, only participants aged ≥8 years who completed a 24‐h dietary recall were included (*n* = 744). It is recognized that children aged 8 years and over have the cognitive ability to recall their own food and beverage intake over the past 24 h.^31^ A further 62 children were excluded for various reasons, leaving a final sample size of 682 (Figure 1).

Ethics approval for the SONIC study was obtained from Deakin University Ethics Committee (ID numbers: EC62‐2009 and HEAG‐H 01_2018), and the Victorian Department of Education and Training (ID numbers: 2011_001151 and 2018_003666). The study was performed in accordance with the ethical standards laid down in the 1964 Declaration of Helsinki. Opt‐in consent for the school to partake in the SONIC study was given by the principal, the primary caregiver provided informed consent for each participant, and written assent was obtained from each child.^29^

### 2.2 Dietary intake data

Participants completed 1 × 24‐h dietary recall individually at school with a researcher, with data representing both school versus non‐school day intake depending on the circumstances of the previous day.^30^ Researchers administering the 24‐h dietary recalls were trained by an Accredited Practising Dietitian.^29^ In Cohort 1 (2010–2013), a three‐pass, face‐to‐face, 24‐h dietary recall method was administered.^30^ In Cohort 2 (2018–2019), recalls were administered by a trained researcher using a newly developed, web‐based, five‐pass dietary assessment tool, the ASA‐24‐Australia‐2016.^32^ This new tool was advantageous as it allowed direct entry of foods recalled by participants into online software. The multiple pass method prompts children to recall whether foods consumed were homemade or commercially prepared (including brand information). For example, children were asked if a food recalled was made from a home recipe, from a restaurant, fast food place, commercially frozen or something else? Foods eaten away from home were recorded using the same standardized prompts as per other foods. In both cohorts, reported food intake was coded to foods contained within the AUSNUT 2011–2013 food composition database.^33^ In Cohort 1, this was done using dietary analysis programme FoodWorks Version 8,^34^ and in Cohort 2, this was automated within the ASA‐23‐Australia‐2016 software.^32^ AUSNUT 2011–2013 is Australia's most comprehensive food composition database containing 5740 unique food items and corresponding nutrient data.^33^ Within this database, each food item is assigned a unique 8‐digit food code, for example, if a participant reported consumption of a commercial chocolate chip biscuit this was coded as ‘13105001: Biscuit, sweet, chocolate chip, commercial.’ If the child reported a homemade version, the item was coded as ‘13105003: Biscuit, sweet, chocolate chip, homemade from basic ingredients, fat not further defined.’ Following the coding of reported food intake into AUSNUT 2011–2013 food codes, a previously established database was utilized to categorize each food item according to NOVA groups.^30^, ^33^, ^35^

The NOVA system defines its four groups as: Group 1—unprocessed or minimally processed foods (none or basic processing applied; for example, grains, such as white rice, fresh vegetables, meat which is whole or in the form of steaks), Group 2—processed culinary ingredients (substances procured directly from unprocessed foods or nature; e.g., sugar, salt, oils, fats), Group 3—processed foods (foods created through the combination of unprocessed foods and processed culinary ingredients; e.g., freshly baked breads, simple cheeses and canned vegetables), Group 4—UPF (formulations of multiple substances requiring complex industrial processing procedures, often including ingredients not found in a household kitchen; e.g., carbonated soft‐drinks, mass‐produced breads, ready‐to‐heat/ready‐to‐eat meals).^4^, ^5^ Detailed methodology related to the development of the previous database which codes AUSNUT 2011–2013 food codes to NOVA categories can be found elsewhere.^35^ In brief each of the 5740 unique food codes contained with AUSNUT 2011–2013 were investigated to code as one of four NOVA groups. This was completed for single food items (e.g., 13105001: biscuit, sweet, chocolate chip, commercial = UPF) and the underlying ingredients contained within culinary preparations, for example, handmade recipes, like ‘13105003: Biscuit, sweet, chocolate chip, homemade from basic ingredients, fat not further defined’ were disaggregated and the individual ingredients (e.g., chocolate, egg, butter, salt, flour, margarine spread, oil and sugar) were separately categorized into NOVA groups.^35^ In the current study, mixed dishes consumed by children and deemed to be a culinary preparation accounted for 65% of all food items in this sample, as per Machado et al.^35^ previous methodology these foods were disaggregated into individual ingredients and then categorized into NOVA groups.

Because recipes were disaggregated into raw ingredients, additional adjustments to account for the weight change of an ingredient as it lost or gained moisture when going from raw to cooked form (e.g., as eaten by study participants) were made. This was completed using published weight change factors available in the AUSNUT 2011–2012 food composition database (see Figure S1).^36^ This process produced a revised energy content value of ingredients, which accounted for the change in energy density of a product going from a raw to cooked form. Further explanation on this adjustment process is available in Supporting Information S1.

The proportion of weight (g) and energy (kJ) consumed from each of the NOVA groups in the total diet was calculated. Analyzing UPF intake by grams accounts for products that may not be captured by energy intake, such as artificially sweetened beverages.^37^ However, examining UPF consumption by energy directly relates to this study's primary outcome of weight status and is consistent with a broad range of literature examining the association between UPF and obesity globally.^11^, ^38^

### 2.3 Anthropometric data

Anthropometric data were collected at school by trained researchers, whilst children were wearing light clothing and without shoes. Protocols were consistent with those used in a previous national survey and detailed elsewhere.^27^ Weight and height were measured using digital scales (Tanita BC‐351) and stadiometers (Charder—HM‐200P Portstad), respectively.^30^ Waist circumference (WC) was measured to the nearest 0.1 cm with a steel tape (Lufkin Executive Thinkline W606PM) at the end of a normal expiration, at the narrowest point between the lower costal border and the top of the iliac crest. Two measurements were taken, and if the difference between these two measures was >10 mm, a third measure was taken. In analysis, the average of two measures was used, or in the case of three available measures, the median was used. Age‐ and sex‐adjusted body mass index (BMI) *z*‐scores^39^; International Obesity Task Force (IOTF) definitions of underweight, healthy weight, overweight and obese^40^; and waist‐to‐height ratio (WtHR; values ≥0.5 denoting having a high WtHR)^41^ were used as indicators of obesity.

### 2.4 Sociodemographic data

Data regarding sociodemographic characteristics was collected via questionnaires sent to parents of participating children. This included information regarding participants' age, sex, the highest educational attainment by a parent or guardian (low SES—some or no high school education; mid SES—technical/trade certificate; high SES—tertiary certificate),^30^ area‐level SES categorized by level of disadvantage (measured by the Socio‐Economic Indexes for Areas Index of Relative Socio‐Economic Disadvantage [IRSD]),^42^ and geographic location of the residence (grouped by Australian Bureau of Statistics classifications of remoteness).^43^

### 2.5 Statistical analysis

As there were no differences in UPF intake (% total energy from UPF in Cohort 1 = 46.7%, Cohort 2 = 48.1%; *p* = 0.503) or key sociodemographic characteristics (sex, age, SES or BMI *z*‐score) between children who participated in 2010–2013 versus 2018–2019, we have combined populations from both cohorts for this analysis (Figure 1). For all analyses, a *p*‐value <0.05 was considered significant.

Data was scrutinized for implausible outliers in comparison to peers within the sample and national data.^44^ The Goldberg cut‐off method, using pediatric age‐ and sex‐specific adjusted cut‐offs, was used to identify potential under‐reporters of energy intake.^45^ Schofield equations were used to estimate the child's basal metabolic rate, and participants were excluded (*n* = 40, Figure 1) if their estimated intake to basal metabolic rate ratio was below the determined Goldberg cut‐off value. A statistical cut‐off of >4 standard deviations (SD) from the cohort mean was used as a cut‐off for over‐reporters of energy intake (*n* = 3).^46^

Descriptive statistics (mean, SD and proportions) were used to explore sample characteristics and UPF intake. Quartiles of UPF intake were determined, and *t*‐tests and chi square analyses (as appropriate) were used to examine sociodemographic characteristics across quartiles. To further examine differences in UPF consumption across sociodemographic characteristics, adjusted linear regression modelling was completed. UPF intake (as both [i] energy kJ/day and proportion of total energy and [ii] grams and proportion of total amount of food consumed) was treated as the continuous dependent variable, and age group (8–9 vs. 10–12 years), sex, geographical location and SES (both IRSD and parent education) were each considered as categorical independent variables in separate regression models. The mean differences between groups within these characteristics were adjusted for each of the characteristics listed excluding the one being analysed (e.g., sex adjusted for age, geographical location and SES‐IRSD). Multiple linear regression was used to test the relationship between a 10% difference in UPF consumption (independent continuous variable) and BMI *z*‐score (dependent continuous variable), whilst logistic regression was used to assess the association between UPF consumption and weight status and WtHR (both dependent binary categorical variables). For each individual indicator of obesity (BMI *z*‐score, underweight and normal weight vs. overweight and obesity, WtHR <0.5 vs. WtHR ≥0.5), Model 1 was unadjusted; Model 2 adjusted for age, sex, geographical location, SES‐IRSD, and whether the 24‐h dietary recall gathered data from a school day or non‐school day; and Model 3 was additionally adjusted for total energy intake. Acknowledging the onset of puberty during the ages of 8–12 years, which is characterized by hormonal changes and shifts in body weight and adipose tissue,^47^ age stratified analyses were completed (8–9 and 10–12 years).^47^ The age cut‐offs align with the commencement of adolescence at 10 years,^48^ and typical patterns of pubertal onset, with 8‐ to 9‐year‐old children generally prepubertal, and 10‐ to 12‐year‐old children more likely to be experiencing puberty‐related physiological changes.^49^, ^50^ All modelling described above was adjusted for school clustering, to account for grouping of students within schools.

## 3 RESULTS

### 3.1 Participant characteristics

The mean age of the children was 10.2 years (SD = 1.2), and over half of the sample were aged 10–12 years (*n* = 393, 58%). Forty‐seven percent (*n* = 318) were female and 16% (*n* = 109) of participants were classified as living with overweight and 4.0% (*n* = 25) with obesity; whilst 24% (*n* = 160) had a high WtHR (Table 1). Seventy‐five percent of dietary recalls captured food consumed on a school day, with the remaining on non‐school days. Most children resided in major cities (73%) and were of high SES (IRSD = 54% high; parent education level = 64% high). In unadjusted analyses, sex and parent education level were the only characteristics demonstrating significant associations with quartiles of proportion of energy from UPF (*p* = 0.05 and *p* = 0.04, respectively, Table 1). Quartile of UPF consumption expressed as a proportion of total amount of food consumed (g) is shown in Table S1. In this unadjusted analysis, no sociodemographic characteristics were significantly associated with UPF intake.

**TABLE 1:** Sociodemographic characteristics and ultra‐processed foods (UPF) consumption of primary schoolchildren from Victoria, Australia (*n* = 682).

Characteristic		Quartile of UPF consumption (% energy from UPF): mean (SD) or proportion (*n*)				
	Overall (*n* = 682)	Q1 (*n* = 167)	Q2 (*n* = 165)	Q3 (*n* = 177)	Q4 (*n* = 173)	*p* Value^a^
Total energy intake (kJ)	8409 (2552)	8416 (2662)	8273 (2483)	8690 (2584)	8244 (2473)	0.53
UPF consumption, % of energy	47.2 (0.7)	23.7 (7.7)	39.0 (3.6)	52.5 (4.3)	72.2 (9.1)	
Age (years)	10.2 (1.2)	10.4 (1.2)	10.2 (1.2)	10.1 (1.3)	10.3 (1.3)	0.19
Sex						0.05
Female	46.6 (318)	44.3 (74)	45.4 (75)	55.4 (98)	41.0 (71)	
Male	53.4 (364)	55.7 (93)	54.6 (90)	44.6 (79)	59.0 (102)	
Geographic location^b^						0.62
Inner regional	23.5 (152)	19.8 (32)	25.5 (39)	24.7 (41)	24.2 (40)	
Major cities	76.5 (494)	80.2 (130)	74.5 (114)	75.3 (125)	75.8 (125)	
SES—IRSD						0.94
Low—most disadvantaged	22.6 (154)	22.2 (37)	23.0 (38)	23.2 (41)	22.0 (38)	
Mid	23.6 (161)	23.3 (39)	20.6 (34)	23.7 (42)	26.6 (46)	
High—least disadvantaged	53.8 (367)	54.5 (91)	56.4 (93)	53.1 (94)	51.4 (89)	
Parent education level^b^						0.04
Low	21.4 (121)	11.4 (16)	27.3 (39)	24.7 (34)	22.1 (32)	
Mid	15.2 (86)	18.6 (26)	13.3 (19)	15.9 (22)	13.1 (19)	
High	63.4 (359)	70.0 (98)	59.4 (85)	59.4 (82)	64.8 (94)	
BMI weight category						0.88
Underweight	6.6 (45)	6.0 (10)	7.3 (12)	5.7 (10)	7.5 (13)	
Healthy weight	73.7 (503)	73.6 (123)	70.3 (116)	75.7 (134)	75.1 (130)	
Overweight	16.0 (109)	17.4 (29)	19.4 (32)	14.1 (25)	13.3 (23)	
Obese	3.7 (25)	3.0 (5)	3.0 (5)	4.5 (8)	4.1 (7)	
WtHR						0.90
WtHR <0.5	76.5 (522)	78.4 (131)	75.8 (125)	76.8 (136)	75.1 (130)	
WtHR ≥0.5	23.5 (160)	21.6 (36)	24.2 (40)	23.2 (41)	24.9 (43)	
BMI *z*‐score	0.22 (1.0)	0.16 (1.0)	0.26 (1.0)	0.24 (1.0)	0.21 (1.0)	0.71
Height (cm)	142.8 (9.6)	143.8 (9.7)	143.3 (9.4)	142.0 (9.8)	142.0 (9.6)	0.16
Weight (kg)	37.3 (9.6)	37.9 (10.4)	37.6 (9.0)	36.9 (10.1)	36.7 (8.8)	0.53
WC (cm)	66.5 (9.3)	66.2 (9.2)	67.4 (9.5)	66.1 (9.8)	66.4 (8.8)	0.37

Abbreviations: BMI, body mass index; IRSD, Index of Relative Socio‐Economic Disadvantage; SES, socioeconomic status; SD, standard deviations; WC, waist circumference; WtHR, waist‐to‐height ratio.

^a^ *p* Values estimated by chi square and *t* test analyses.

^b^ Geographic location *n* = 646; parent education level *n* = 566.

### 3.2 NOVA group and subgroup intakes

All children consumed some UPF on the day of the 24‐h dietary recall, and just under half of all energy consumed was obtained from UPF (47.2%) (Table 2). Unprocessed or minimally processed foods contributed 32.3% of total energy intake, whilst processed culinary ingredients and processed foods provided 6.8% and 13.7%, respectively (Table 2). The top five sources of energy from UPF were pastries, buns and cakes (4.8%), biscuits (4.7%), confectionery (4.5%), breakfast cereals (4.1%) and fast food dishes (3.9%).

**TABLE 2:** Energy intake (kJ) and proportion of total energy intake (%) of foods from NOVA groups and subgroups.^4^

NOVA group	Subgroup name	Mean energy intake (kJ)	SD	Mean proportion of total energy intake (%)	SD
Unprocessed/minimally processed foods	*Unprocessed/minimally processed foods*	2715.3	63.5	32.3	0.6
	Cereals	602.6	35.2	6.8	0.4
	Milk and plain yoghurt	530.8	20.1	6.4	0.2
	Fruits	402.9	15.5	5.1	0.2
	Poultry	272.4	22.0	3.2	0.2
	Red meat	249.2	18.3	3.0	0.2
	Pasta	230.9	19.9	2.8	0.2
	Vegetables	169.9	9.3	2.1	0.1
	Potatoes/tubers	81.8	7.6	1.0	0.1
	Eggs	67.8	5.8	0.8	0.1
	Fish	45.7	8.5	0.5	0.1
	Legumes	28.6	3.6	0.3	0.0
	Nuts and seeds	30.0	12.3	0.3	0.1
	Other^a^	2.6	0.3	0.0	0.0
	Freshly squeezed juices	0.2	0.1	0.0	0.0
Processed culinary ingredients	*Processed culinary ingredients*	596.7	24.8	6.8	0.2
	Plant oil	242.2	11.7	2.8	0.1
	Animal fats	212.6	12.9	2.4	0.1
	Table sugar	112.0	9.8	1.2	0.1
	Other^b^	29.8	5.1	0.4	0.1
	Salt	0.0	0.0	0.0	0.0
Processed foods	*Processed foods*	1138.7	40.0	13.7	0.4
	Processed breads	710.0	29.9	8.7	0.3
	Cheese	237.5	14.9	2.8	0.2
	Bacon and other salted/canned/smoked meat	79.9	10.2	1.0	0.1
	Other^c^	83.6	14.1	0.9	0.1
	Vegetables and other plant foods	27.6	4.0	0.3	0.0
	Beer and wine	0.1	0.1	0.0	0.0
Ultra‐processed foods	*Ultra‐processed foods*	3958.3	76.2	47.2	0.7
	Pastries, buns and cakes	435.0	36.8	4.8	0.4
	Biscuits	387.6	22.5	4.7	0.3
	Confectionary	383.5	22.7	4.5	0.2
	Breakfast cereals	320.1	16.1	4.1	0.2
	Fast food dishes	330.7	32.4	3.9	0.4
	Mass‐produced packaged breads	282.3	19.8	3.5	0.3
	Sausage/other reconstituted meat products	242.0	21.6	2.8	0.2
	Fruit drinks and iced teas	215.7	12.8	2.7	0.2
	Packaged salty snacks	203.5	19.0	2.3	0.2
	Ice cream/frozen yoghurts/ice pops	193.0	15.9	2.3	0.2
	Packaged ready meals	186.7	21.0	2.3	0.3
	Milk‐based drinks	163.2	16.8	1.9	0.2
	Sauces, dressing and gravies	140.5	12.0	1.6	0.1
	Margarine/other spreads	133.8	8.1	1.6	0.1
	Soft‐drinks, carbonated	118.8	10.5	1.4	0.1
	Instant/canned soups	85.3	14.8	1.1	0.2
	Industrial desserts	47.4	8.7	0.5	0.1
	Ultra‐processed cheese	42.1	5.1	0.5	0.1
	Industrial French fries	37.9	9.7	0.4	0.1
	Other^d^	5.9	2.5	0.1	0.0
	Frozen pizza	2.1	2.1	0.0	0.0
	Alcoholic distilled drinks	1.2	1.1	0.0	0.0

Abbreviation: SD, standard deviations.

^a^ Including non‐flavoured coffee and tea.

^b^ Including vinegar; baking soda.

^c^ Including salted or sugared nuts and seeds.

^d^ Including soya; baby formula.

### 3.3 Associations between UPF intake and age, sex, geographic location and SES

Analysing the absolute energy intake of UPF across sociodemographic groups, Tables 3 and 4 show that after adjustment for key covariates (age, geographic location and SES [IRSD]), boys consumed 425 kJ/day more energy from UPF than girls (*p* = 0.01). Additionally, there was a non‐significant trend that participants from inner regional areas consumed 361 kJ/day more energy from UPF than those from major cities (*p* = 0.06). There was no association between UPF consumption and age group or SES as defined by IRSD. When considering parent education level as a measure of SES, the low SES group consumed 678 kJ/day more energy from UPF than participants from high SES backgrounds (*p* = 0.03) (Table 4). However, after adjustment for overall energy intake, none of the above associations remained significant. Fewer significant associations were seen when measuring UPF as an absolute amount or proportion of total grams consumed. Whilst girls had a 3% lower proportion of grams of UPF in the diet (approximately 77 g/day), these observations were no longer significant after adjustment for covariates (Table 3).

**TABLE 3:** Intake of ultra‐processed foods (UPF; energy [kJ], g, % of daily energy and % of daily grams) by sociodemographic characteristics (age group, sex and geographic location).

UPF consumption	Unadjusted values					Adjusted values^a^				
	Mean	Robust SE	Mean	Robust SE	*p* Value	Mean	Robust SE	Mean	Robust SE	*p* Value
Age group	8–9 years		10–12 years			8–9 years		10–12 years		
Energy (kJ/day)	4068	117	3878	93	0.20	4122	34	3850	37	0.09
Proportion of total energy (%)	48	1	46	1	0.26	48	1	46	1	0.23
Amount (g/day)	555	23	545	20	0.68	559	25	540	21	0.42
Proportion of total amount of food consumed (%)	30	1	29	1	0.14	30	1	28	1	0.08

Sex	Boys		Girls			Boys		Girls		
Energy (kJ/day)	**4122**	**115**	**3771**	**108**	**0.04**	**4163**	**126**	**3738**	**97**	**0.01**
Proportion of total energy (%)	47	1	47	1	0.93	47	1	47	1	0.85
Amount (g/day)	**585**	**24**	**508**	**22**	**0.01**	588	24	502	25	0.07
Proportion of total amount of food consumed (%)	**31**	**1**	**28**	**1**	**0.05**	31	1	27	1	0.22

Geographic location	Major cities		Inner regional			Major cities		Inner regional		
Energy (kJ/day)	3927	92	4100	147	0.32	3880	103	4241	139	0.06
Proportion of total energy (%)	47	1	48	1	0.55	49	1	47	1	0.23
Amount (g/day)	542	22	568	33	0.51	540	25	574	45	0.07
Proportion of total amount of food consumed (%)	29	1	29	1	0.83	29	1	30	1	0.22

Abbreviation: SE, standard error.

^a^ Estimates adjusted for: age, sex, geographic location, socioeconomic status (Index of Relative Socio‐Economic Disadvantage) using regression analysis; excluding the respective characteristic being analysed.

**TABLE 4:** Intake of ultra‐processed foods (UPF energy [kJ]), g, % of daily energy and % of daily grams) by sociodemographic characteristics (socioeconomic status [SES] and geographic location).

UPF consumption	Unadjusted values							Adjusted values^a^						
	Mean	Robust SE	Mean	Robust SE	Mean	Robust SE	*p* Value	Mean	Robust SE	Mean	Robust SE	Mean	Robust SE	*p* Value
	Low SES		Mid SES		High SES			Low SES		Mid SES		High SES		
SES (IRSD)														
Energy (kJ/day)	3927	76	4181	271	3874	83	0.55	3804	138	4266	271	3901	88	0.25
Proportion of total energy (%)	46	2	49	3	47	1	0.72	46	1	49	3	47	1	0.41
Amount (g/day)	567	28	549	54	542	21	0.76	549	52	565	56	540	24	0.92
Proportion of total amount of food consumed (%)	29	1	30	3	29	1	0.84	29	2	31	3	29	1	0.84
SES (parent education level)														
Energy (kJ/day)	4433	203	3839	272	3832^c^	82	0.04	4506	208	3816	282	3828	93^c^	0.03
Proportion of total energy (%)	50	2	46	3	46	1	0.19	50	2	46	3	46	1	0.14
Amount (g/day)	618	39	564	47	519	23	0.06	621	41	555	47	520	25	0.10
Proportion of total amount of food consumed (%)	33	2	30	2	28	1	0.07	33	2	30	3	28	1	0.07

Abbreviation: IRSD, Index of Relative Socio‐Economic Disadvantage.

^a^ Estimates adjusted for: age, sex, geographic location, SES (IRSD) using regression analysis; excluding the respective characteristic being analysed.

### 3.4 Associations between UPF intake and indicators of obesity

Including all children there were no significant associations between any indicator of obesity and intake of UPF expressed as a 10% difference in the proportion of daily energy intake (kJ) or amount of food consumed (g) (Table 5). When stratified by age group, results varied. Among 8‐ to 9‐year‐old children, no associations were found between UPF intake and any indicators of obesity. Conversely, among those aged 10–12 years, there was a significant positive association between the proportion of UPF consumed in grams and BMI *z*‐score and abdominal obesity. After adjustment for age, sex, SES, geographic location and day type of 24‐h recall, each 10% increment in the proportion of UPF consumed (g) was associated with a 0.07 unit (95% CI 0.01, 0.12) higher BMI *z*‐score and a 19% increase in the odds of central obesity (odds ratio [OR] 1.19, 95% CI 1.07, 1.33) (Table 5). This association remained following additional adjustment for energy intake. There was no association between the proportion of UPF consumed in grams and overweight/obesity. When modelled as the percentage of UPF in the diet as a proportion of energy intake, there were no associations with any indicators of obesity (Table 5).

**TABLE 5:** Associations between 10% increase in percentage of daily energy (% kJ/day) and percentage of daily grams (% g/day) sourced from ultra‐processed foods (UPF) and indicators of obesity (*n* = 646).

10% increase in % kJ/day from UPF	All children (*n* = 646)		7–9 years (*n* = 273)		10–12 years (*n* = 373)	
	*β*‐coefficient (95% CI)	*p*‐Value	*β*‐coefficient (95% CI)	*p*‐Value	*β*‐coefficient (95% CI)	*p*‐Value
BMI *z*‐score						
Model 1	0.007 (−0.03, 0.05)	0.75	−0.01 (−0.08, 0.05)	0.73	0.02 (−0.03, 0.06)	0.45
Model 2	0.002 (−0.04, 0.05)	0.94	−0.02 (−0.09, 0.06)	0.61	0.01 (−0.04, 0.06)	0.63
Model 3	0.003 (−0.04, 0.05)	0.91	−0.02 (−0.09, 0.05)	0.53	0.01 (−0.04, 0.07)	0.58

Overweight/obese (IOTF standard)	OR (95% CI)	*p*‐Value	OR (95% CI)	*p*‐Value	OR (95% CI)	*p*‐Value
Model 1	0.95 (0.87, 1.04)	0.26	0.89 (0.78, 1.01)	0.08	0.99 (0.88, 1.11)	0.86
Model 2	0.94 (0.86, 1.03)	0.18	0.86 (0.74, 1.01)	0.06	0.98 (0.87, 1.09)	0.71
Model 3	0.94 (0.86, 1.03)	0.17	0.86 (0.74, 1.01)	0.06	0.98 (0.88, 1.09)	0.69

Abdominal obesity (WtHR ≥0.5)						
Model 1	1.03 (0.94, 1.03)	0.53	0.96 (0.83, 1.10)	0.55	1.07 (0.96, 1.20)	0.22
Model 2	1.02 (0.94, 1.12)	0.59	0.93 (0.80, 1.09)	0.34	1.07 (0.96, 1.20)	0.22
Model 3	1.02 (0.95, 1.12)	0.59	0.93 (0.80, 1.07)	0.30	1.07 (0.96, 1.20)	0.24

10% increase in % g/day from UPF	*β*‐coefficient (95% CI)	*p*‐Value	*β*‐coefficient (95% CI)	*p*‐Value	*β*‐coefficient (95% CI)	*p*‐Value
BMI *z*‐score						
Model 1	0.039 (−0.01, 0.08)	0.09	−0.02 (−0.09, 0.05)	0.57	0.08 (0.03, 0.13)	0.004
Model 2	0.033 (−0.02, 0.08)	0.17	−0.02 (−0.09, 0.05)	0.56	0.07 (0.01, 0.12)	0.02
Model 3	0.031 (−0.02, 0.08)	0.20	−0.03 (−0.09, 0.04)	0.42	0.07 (0.01, 0.12)	0.02

Overweight/obese (IOTF standard)	OR (95% CI)	*p*‐Value	OR (95% CI)	*p*‐Value	OR (95% CI)	*p*‐Value
Model 1	0.97 (0.88, 1.07)	0.53	0.87 (0.72, 1.04)	0.13	1.03 (0.93, 1.15)	0.54
Model 2	0.97 (0.88, 1.07)	0.55	0.85 (0.71, 1.05)	0.13	1.04 (0.92, 1.17)	0.51
Model 3	0.97 (0.88, 1.07)	0.55	0.86 (0.71, 1.04)	0.11	1.04 (0.93, 1.17)	0.51

Abdominal obesity (WtHR ≥0.5)						
Model 1	1.08 (0.98, 1.19)	0.12	0.93 (0.78, 1.10)	0.40	1.17 (1.05, 1.31)	0.004
Model 2	1.10 (0.99, 1.21)	0.07	0.92 (0.78, 1.11)	0.42	1.19 (1.07, 1.33)	0.001
Model 3	1.10 (0.99, 1.21)	0.07	0.92 (0.77, 1.09)	0.32	1.19 (1.07, 1.33)	0.001

*Note*: Model 1—unadjusted. Model 2—Model 1 + sex, age, geographic location, socioeconomic status (Index of Relative Socio‐Economic Disadvantage), type of day of 24‐h dietary recall (school‐day vs. non‐school day). Model 3—Model 2 + total energy intake (kJ). All models used robust standard errors to adjust for clustering of participants within schools.

Abbreviations: BMI, body mass index; CI, confidence interval; IOTF, International Obesity Task Force; OR, odds ratio; WtHR, waist‐to‐height ratio.

Model 2 and 3: adjustment for geographic location reduced sample size to *n* = 646, due to missing data.

## 4 DISCUSSION

This cross‐sectional analysis of the association between UPF intake and sociodemographic characteristics—as well as indicators of obesity—in primary schoolchildren revealed that almost half of participants' diets (47.2% of total energy) consisted of UPF. Boys and those with parents of low education consumed more absolute energy from UPF than girls and those from parents with higher education levels, respectively. Findings from previous studies regarding sex differences in UPF intake are generally inconsistent,^8^, ^10^ higher intakes of UPF in boys may be due to their higher energy requirements and greater absolute total energy intake.^51^ Contrastingly, there is consistent evidence to indicate that children from lower SES backgrounds consume more UPF than their higher SES peers.^8^, ^10^

When all children were analysed together, this study revealed no significant associations between the proportion of total energy or grams from UPF and overweight/obesity, BMI *z*‐score or WtHR. This finding is consistent with many other cross‐sectional studies, which commonly report no association between UPF intake and adiposity in children.^21^, ^25^ When stratified by age group, there was evidence of a positive association between the proportion of UPF in the diet among older children, aged 10–12 years, but no associations in the younger group aged 8–9 years. Specifically, among 10‐ to 12‐year‐old children, a higher proportion of UPF in the diet (as expressed as percent of total grams of food intake per day) was associated with a modest increase in BMI *z*‐score (0.07 units) and 19% higher odds of abdominal obesity, whilst no association was found with the overweight/obesity category. The finding related to a BMI *z*‐score contrasts with the most recent systematic review of observational studies on this topic, in which all seven included cross‐sectional studies reported null or inverse associations between UPF intake and BMI *z*‐score among children of various ages. Whilst statistically significant, the magnitude of the association observed in our study is small and may lack clinical significance. An increase in autonomy and other reward‐related systems may influence dietary intake differently among 10‐ to 12‐year‐old children compared to younger children.^52^ In relation to abdominal obesity, our findings align with two past studies conducted in adolescents.^10^, ^53^ Firstly, Neri et al.^10^ reported that a 10% increase in UPF intake (as a proportion of total grams), as measured by repeated (up to 2) 24‐h dietary recalls, was associated with greater odds of having abdominal overweight/obesity (OR 1.07; 95% CI 1.01–1.13), in their cross‐sectional analysis of a large (*n* = 3587), nationally representative sample of US adolescents aged 12–19 years (2017–2018 National Health and Nutrition Examination Survey [NHANES]).^10^ Similarly, a cross‐sectional study of 576 adolescents aged 10–17 years from Brazil reported a positive association between UPF intake, assessed by a food frequency questionnaire and abdominal obesity.^53^ However, many other cross‐sectional studies examining the association between UPF consumption and overweight/obesity have reported null findings or inverse associations,^25^ with the majority of these being conducted in Brazil. Across these studies, there are notable methodological discrepancies related to the age of participants, the dietary assessment tools used to measure UPF intake and adjustment for potential confounders.

Positive associations between UPF intake and obesity in children are more consistently reported in longitudinal studies.^8^, ^21^, ^23^, ^54^ In a sample of 9025 British children, increased UPF intake at 7 years of age predicted a greater BMI *z*‐score and WC at 24 years of age,^8^ whilst a significant increase in WC at 8 years for every 10% increase in UPF intake at 4 years was found in a sample of 500 Brazilian children.^54^ Therefore, De Amicis et al. posited that the body composition of children is influenced by consistently high UPF consumption over time,^21^ which is better captured by longitudinal study designs.

Although many biological mechanisms have been proposed to explain how UPF contribute to obesity, the association is not fully understood.^9^ The increased energy density of these foods may play a role in increasing ad libitum energy intake, with UPF shown to be considerably more energy dense than unprocessed foods in the Australian food supply.^35^ Additionally, UPF are shown to be less satiating than unprocessed foods, which can also increase ad libitum intake.^9^ Further, even when a child is in a satiated state, highly palatable foods (e.g., UPF) still promote food‐seeking behaviours—potentially leading to hyperenergetic diets.^55^ Another proposed mechanism potentially mediating this association involves the textural and structural alterations of the food matrix that occur with ultra‐processing, thus changing nutrient bioavailability whilst incorporating additives and contaminants.^56^, ^57^

With UPF comprising almost 50% of Australian children's total energy intake, (which tend to be high in free sugars, sodium, saturated and trans fats and energy density) they are likely to be displacing unprocessed or minimally processed foods from the diet.^11^, ^35^ According to the Australian Dietary Guidelines, unprocessed foods should form the basis of children's diets and are crucial for their healthy growth and development.^58^ Critically, these foods were responsible for only one third of energy intake in this sample. Further longitudinal investigation is required in Australian children to better understand the role UPF play on children's anthropometrics.

This was the first study examining the association between UPF intake, sociodemographic characteristics and indicators of obesity in Australian children. There are several key strengths present in our study, including a large sample size. The use of a 24‐h dietary recall to gather dietary intake data was consistent with national surveys and followed gold standard guidelines.^59^ However, we acknowledge that a single recall is limited and does not provide accurate data on an individual's usual intake. The classification of dietary data according to the NOVA system onto this data was very thorough, utilizing a previous method that included the disaggregation of recipes to allow for classification of individual ingredients rather than whole food items.^35^ Additionally, this method's accuracy was further increased via the adjustment for weight change factors. Also, this study examined associations between multiple indicators of obesity with UPF. Finally, by including analysis of UPF intake measured as both a proportion of total weight of food and as total energy intake consumed per day, this study is highly relevant and comparable to current and future research in the field.^25^

However, several limitations should also be considered. First, the cross‐sectional study design, which does not account for continued consumption patterns over time, may have prevented a finding regarding the association between UPF and obesity.^21^ Second, the convenience sample selection and low response rate may limit the generalizability of our findings to the broader population. Third, recall bias may have impacted 24‐h dietary data collection; however, inconsistencies were minimized by using the multiple pass method and the dietary recall being assisted by trained researchers. Fourth, no data was available on additional confounders (e.g., physical activity), which would have been important to consider. Fifth, the sample was heavily skewed towards high SES participants (despite large recruitment from public schools), and the proportion of children with obesity was lower than the national average,^2^ which may have biased the results. Further, it is acknowledged that the method of dietary recall slightly changed across cohorts, although both were interviewer‐administered, and this likely had minimal impact.

## 5 CONCLUSION

This study found that in primary schoolchildren from Victoria, Australia, UPF comprised almost half of total energy intake. Furthermore, in this sample of children, sex and SES significantly impacted the absolute energy intake sourced from UPF—however, this was not true for UPF consumption as a proportion of total energy intake. Findings related to UPF intake and indicators of obesity varied by age, with no associations observed in children aged 8–9 years, and a small positive association between the proportion of UPF in the diet and BMI *z*‐score and abdominal obesity in children aged 10–12 years. Further longitudinal research in Australian pediatric samples is required to understand the potential impact of UPF intake on adiposity outcomes and how this may vary across stages of childhood. Overall, the level of UPF consumption in this sample was remarkably high. Furthermore, the low intake of unprocessed or minimally processed foods indicates a need for targeted interventions towards primary schoolchildren in Australia—aiming to improve knowledge around, and decrease consumption of, these potentially harmful products.

## AUTHOR CONTRIBUTIONS

LC, CG, KAB and KEL contributed to the design of the study. LC conducted the analysis with major support from KL and PPM. LC wrote the manuscript with major editing by CG. All authors provided input into the interpretation of data and have read, reviewed and approved the final manuscript.

## CONFLICT OF INTEREST STATEMENT

No conflict of interest was declared.

## Data Availability

The data used in this study cannot be made publicly available as participants did not provide consent for their data to be used for purposes other than described in the original study aims.

## References

[1] World Health Organisation . Overweight and obesity. Updated June 9 2021. Accessed April 22, 2022. https://www.who.int/news-room/fact-sheets/detail/obesity-and-overweight

[2] Australian Institute of Health & Welfare . Australia's children. AIHW. Updated February 25 2022. Accessed July 11, 2023. https://www.aihw.gov.au/reports/children-youth/australias-children

[3] Reilly JJ . Descriptive epidemiology and health consequences of childhood obesity. Best Pract Res Clin Endocrinol Metab. 2005;19(3):327‐341. doi:10.1016/j.beem.2005.04.002 16150378

[4] Monteiro CA , Cannon G , Lawrence M , Louzada ML , Pereira Machado P . Ultra‐processed foods, diet quality, and health using the NOVA classification system. FAO; 2019.

[5] Monteiro CA , Cannon G , Levy RB , et al. Ultra‐processed foods: what they are and how to identify them. Public Health Nutr. 2019;22(5):936‐941. doi:10.1017/S1368980018003762 30744710 PMC10260459

[6] Askari M , Heshmati J , Shahinfar H , Tripathi N , Daneshzad E . Ultra‐processed food and the risk of overweight and obesity: a systematic review and meta‐analysis of observational studies. Int J Obes (Lond). 2020;44(10):2080‐2091. doi:10.1038/s41366-020-00650-z 32796919

[7] Baker P , Machado P , Santos T , et al. Ultra‐processed foods and the nutrition transition: global, regional and national trends, food systems transformations and political economy drivers. Obes Rev. 2020;21(12):e13126. doi:10.1111/obr.13126 32761763

[8] Chang K , Khandpur N , Neri D , et al. Association between childhood consumption of Ultraprocessed food and adiposity trajectories in the Avon longitudinal study of parents and children birth cohort. JAMA Pediatr. 2021;175(9):e211573. doi:10.1001/jamapediatrics.2021.1573 34125152 PMC8424476

[9] Hall KD , Ayuketah A , Brychta R , et al. Ultra‐processed diets cause excess calorie intake and weight gain: an inpatient randomized controlled trial of ad libitum food intake. Cell Metab. 2019;30(1):67‐77.e3. doi:10.1016/j.cmet.2019.05.008 31105044 PMC7946062

[10] Neri D , Martínez‐Steele E , Khandpur N , Levy R . Associations between ultra‐processed foods consumption and indicators of adiposity in US adolescents: cross‐sectional analysis of the 2011‐2016 National Health and Nutrition Examination survey. J Acad Nutr Diet. 2022;122:1474‐1487.e2. doi:10.1038/s41366-020-00650-z 35051632

[11] Neri D , Steele EM , Khandpur N , et al. Ultraprocessed food consumption and dietary nutrient profiles associated with obesity: a multicountry study of children and adolescents. Obes Rev. 2022;23(S1):e13387. doi:10.1111/obr.13387 34889015

[12] The Israeli Ministry of Health . Nutritional recommendations. 2019. Accessed March 1, 2023. https://efsharibari.health.gov.il/media/1906/nutritional-recommendations-2020.pdf

[13] Ministry of Health Malaysia . Malaysian dietary guidelines. 2020. Accessed March 1, 2023. https://hq.moh.gov.my/nutrition/wp‐content/uploads/2024/03/latest‐01.Buku‐MDG‐2020_12Mac2024.pdf

[14] Monteiro CA , Cannon G , Moubarac J‐C , et al. Dietary guidelines to nourish humanity and the planet in the twenty‐first century. A blueprint from Brazil. Public Health Nutr. 2015;18(13):2311‐2322. doi:10.1017/S1368980015002165 26205679 PMC10271430

[15] Food and Agriculture Organization . Food‐based dietary guidelines [Internet]. United Nations. 2023 Accessed March 1, 2023. https://www.fao.org/nutrition/education/food‐dietary‐guidelines/regions/en/

[16] Moradi S , Entezari MH , Mohammadi H , et al. Ultra‐processed food consumption and adult obesity risk: a systematic review and dose‐response meta‐analysis. Crit Rev Food Sci Nutr. 2021;63:249‐260. doi:10.1080/10408398.2021.1946005 34190668

[17] Adams J , White M . Characterisation of UK diets according to degree of food processing and associations with socio‐demographics and obesity: cross‐sectional analysis of UK National Diet and Nutrition Survey (2008–12). Int J Behav Nutr Phys. 2015;12(1):160. doi:10.1186/s12966-015-0317-y PMC4683717 26684833

[18] Marino M , Puppo F , Del Bo' C , et al. A systematic review of worldwide consumption of ultra‐processed foods: findings and criticisms. Nutrients. 2021;13(8):2778. doi:10.3390/nu13082778 34444936 PMC8398521

[19] Pagliai G , Dinu M , Madarena MP , Bonaccio M , Iacoviello L , Sofi F . Consumption of ultra‐processed foods and health status: a systematic review and meta‐analysis. Br J Nutr. 2021;125(3):308‐318. doi:10.1017/S0007114520002688 32792031 PMC7844609

[20] Bleiweiss‐Sande R , Sacheck JM , Chui K , Goldberg JP , Bailey C , Evans EW . Processed food consumption is associated with diet quality, but not weight status, in a sample of low‐income and ethnically diverse elementary school children. Appetite. 2020;151:104696. doi:10.1016/j.appet.2020.104696 32251765 PMC7528044

[21] De Amicis R , Mambrini SP , Pellizzari M , et al. Ultra‐processed foods and obesity and adiposity parameters among children and adolescents: a systematic review. Eur J Nutr. 2022;61(5):2297‐2311. doi:10.1007/s00394-022-02873-4 35322333 PMC8942762

[22] Oliveira T , Ribeiro I , Jurema‐Santos G , et al. Can the consumption of ultra‐processed food Be associated with anthropometric indicators of obesity and blood pressure in children 7 to 10 years old? Foods. 2020;9(11):1567. doi:10.3390/foods9111567 33126771 PMC7692221

[23] Costa CS , Del‐Ponte B , Formoso Assuncao MC , Santos IS . Consumption of ultra‐processed foods and body fat during childhood and adolescence: a systematic review. Public Health Nutr. 2018;21(1):148‐159. doi:10.1017/S1368980017001331 28676132 PMC10260745

[24] Petridi E , Karatzi K , Magriplis E , Charidemou E , Philippou E , Zampelas A . The impact of ultra‐processed foods on obesity and cardiometabolic comorbidities in children and adolescents: a systematic review. Nutr Rev. 2024;10(82):913‐928. 10.1093/nutrit/nuad095 37550263

[25] Robles B , Mota‐Bertran A , Saez M , Solans M . Association between ultraprocessed food consumption and excess adiposity in children and adolescents: a systematic review. Obes Rev. 2023;25:e13796. 10.1111/obr.13796 38956887

[26] Petridi E , Karatzi K , Magriplis E , Charidemou E , Philippou E , Zampelas A . The impact of ultra‐processed foods on obesity and cardiometabolic comorbidities in children and adolescents: a systematic review. Nutr Rev. 2024;82(7):913‐928. doi:10.1093/nutrit/nuad095 37550263

[27] Marchese L , Livingstone KM , Woods JL , Wingrove K , Machado P . Ultra‐processed food consumption, socio‐demographics and diet quality in Australian adults. Public Health Nutr. 2022;25(1):94‐104. doi:10.1017/s1368980021003967 34509179 PMC8825971

[28] Louzada MLC , Baraldi LG , Steele EM , et al. Consumption of ultra‐processed foods and obesity in Brazilian adolescents and adults. Prev Med. 2015;81:9‐15. doi:10.1016/j.ypmed.2015.07.018 26231112

[29] Grimes C , Bolton KA , Trieu K , et al. Evaluation of a state‐wide intervention on salt intake in primary schoolchildren living in Victoria, Australia. Public Health Nutr. 2023;26:1456‐1467. doi:10.1017/s1368980023000332 36785876 PMC10346046

[30] Grimes CA , Baxter JR , Campbell KJ , et al. Cross‐sectional study of 24‐hour urinary electrolyte excretion and associated health outcomes in a convenience sample of Australian primary schoolchildren: the Salt and Other Nutrients in Children (SONIC) study protocol. JMIR Res Protoc. 2015;4(1):e7. doi:10.2196/resprot.3994 25592666 PMC4319086

[31] Livingston MB , Robson PJ . Measurement of dietary intake in children. Proc Nutr Soc. 2000;59(2):279‐293. 10946797 10.1017/s0029665100000318

[32] National Cancer Institute . ASA24 – Australia [Internet]. Updated Decemeber 14, 2021. Accessed May 11, 2022. https://epi.grants.cancer.gov/asa24/respondent/australia.html

[33] Food Standards Australia New Zealand . AUSNUT 2011–2013 [Internet]. Updated November 13, 2020. Accessed May 4, 2022. https://www.foodstandards.gov.au/science/monitoringnutrients/ausnut/pages/default.aspx

[34] Xyris Pty Ltd . Foodworks v8.0. Accessed June 3, 2025. https://support.xyris.com.au/hc/en‐us/articles/204455229‐Download‐previous‐versions‐of‐FoodWorks‐Versions‐10‐9‐8‐7‐6‐5‐4‐and‐3

[35] Machado PP , Steele EM , Levy RB , et al. Ultra‐processed foods and recommended intake levels of nutrients linked to non‐communicable diseases in Australia: evidence from a nationally representative cross‐sectional study. BMJ Open. 2019;9(8):e029544. doi:10.1136/bmjopen-2019-029544 PMC6720475 31462476

[36] Food Standards Australia New Zealand . Weight change factors [Internet]. Updated September 15, 2020. Accessed June 8, 2023. https://www.foodstandards.gov.au/industry/npc/Pages/weight‐change‐factors.aspx

[37] Beslay M , Srour B , Méjean C , et al. Ultra‐processed food intake in association with BMI change and risk of overweight and obesity: a prospective analysis of the French NutriNet‐santé cohort. PLoS Med. 2020;17(8):e1003256. doi:10.1371/journal.pmed.1003256 32853224 PMC7451582

[38] Machado PP , Steele EM , Levy RB , et al. Ultra‐processed food consumption and obesity in the Australian adult population. Nutr Diabetes. 2020;10(1):39. doi:10.1038/s41387-020-00141-0 33279939 PMC7719194

[39] Kuczmarsk RJ , Ogden CL , Guo SS , et al. CDC Growth Charts for the United States: methods and development. Vital Health Stat. 2002; 246:1‐190. 12043359

[40] Cole TJ , Bellizzi MC , Flegal KM , Dietz WH . Establishing a standard definition for child overweight and obesity worldwide: international survey. BMJ. 2000;320(7244):1240‐1243. doi:10.1136/bmj.320.7244.1240 10797032 PMC27365

[41] Garnett S , Baur L , Cowell C . Waist‐to‐height ratio: a simple option for determining excess central adiposity in young people. Int J Obes (Lond). 2008;32(6):1028‐1030. 18414423 10.1038/ijo.2008.51

[42] Australian Bureau of Statistics . Census of population and housing: socio‐economic indexes for areas (SEIFA). Cat. 2033.0.55.001. ABS. Updated March 27, 2018. Accessed July 11, 2022. https://www.abs.gov.au/ausstats/abs@.nsf/Lookup/by%20Subject/2033.0.55.001~2016~Main%20Features~SOCIO‐ECONOMIC%20INDEXES%20FOR%20AREAS%20(SEIFA)%202016~1

[43] Australian Bureau of Statistics . Australian statistical geography standard (ASGS). Cat 1270.0.55.005 [Internet]. ABS. Updated March 16, 2018. Accessed July 11, 2022. https://www.abs.gov.au/ausstats/abs@.nsf/Latestproducts/1270.0.55.005Main%20Features1July%202016?opendocument&tabname=Summary&prodno=1270.0.55.005&issue=July%202016&num=&view

[44] Australian Bureau of Statistics . Australian health survey: nutrition first results – foods and nutrients. Commonwealth Government. Updated May 9, 2014. Accessed August 8, 2022. https://www.abs.gov.au/statistics/health/health‐conditions‐and‐risks/australian‐health‐survey‐nutrition‐first‐results‐foods‐and‐nutrients/latest‐release

[45] Black A . Critical evaluation of energy intake using the Goldberg cut‐off for energy intake:basal metabolic rate. A practical guide to its calculation, use and limitations. Int J Obes (Lond). 2000;24(9):1119‐1130. doi:10.1038/sj.ijo.0801376 11033980

[46] Grimes CA , Riddell LJ , Campbell KJ , et al. Dietary intake and sources of sodium and potassium among Australian schoolchildren: results from the crosssectional salt and other nutrients in children (SONIC) study. BMJ Open. 2017;7(10):1‐10. 10.1136/bmjopen-2017-016639 PMC5665305 29084791

[47] Abreu AP , Kaiser UB . Pubertal development and regulation. LancetDiabetes Endocrinol. 2016;4:254‐264. doi:10.1016/S2213-8587(15)00418-0 PMC5192018 26852256

[48] World Health Organization . Adolescent health. Accessed March 10, 2024. https://www.who.int/health‐topics/adolescent‐health#tab=tab_1

[49] Emmanuel M , Bokor BR . Tanner Stages. StatPearls Publishing; 2022. 29262142

[50] Karpati AK , Rubin CH , Kieszak SM , Marcus M , Troiano RP . Stature and pubertal stage assessment in American boys: the 1988–1994 third National Health and nutrition examination survey. J Adolesc Health. 2002;30:205‐212. 11869928 10.1016/s1054-139x(01)00320-2

[51] Torun B . Energy requirements of children and adolescents. Public Health Nutr. 2005;8(7a):968‐993. doi:10.1079/phn2005791 16277815

[52] Neufield LM , Andrade E , Suleiman A , et al. Food choice in transition: adolescent autonomy, agency, and the food environment. Lancet. 2022;399:185‐197. 34856191 10.1016/S0140-6736(21)01687-1

[53] de Souza SF , Conceicao‐Machado MEP , Costa P , et al. Degree of food processing and association with overweight and abdominal obesity in adolescents. Einstein. 2022;30:eAO6619. 10.31744/einstein_journal/2022AO6619 PMC9094610 35584445

[54] Costa C , Rauber F , Leffa PS , Sangalli C , Campagnolo P , Vitolo MR . Ultra‐processed food consumption and its effects on anthropometric and glucose profile: a longitudinal study during childhood. Nutr Metab Cardiovasc Dis. 2019;29(2):177‐184. doi:10.1016/j.numecd.2018.11.003 30660687

[55] Vedovato GM , Vilela S , Severo M , Rodrigues S , Lopes C , Oliveira A . Ultra‐processed food consumption, appetitive traits and BMI in children: a prospective study. Br J Nutr. 2021;125(12):1427‐1436. doi:10.1017/S0007114520003712 32962770

[56] Srour B , Kordahi MC , Bonazzi E , Deschasaux‐Tanguy M , Touvier M , Chassaing B . Ultra‐processed foods and human health: from epidemiological evidence to mechanistic insights. Lancet Gastroenterol Hepatol. 2022;7(12):1128‐1140. doi:10.1016/S2468-1253(22)00169-8 35952706

[57] Dicken SJ , Batterham RL . The role of diet quality in mediating the association between ultra‐processed food intake, obesity and health‐related outcomes: a review of prospective cohort studies. Nutrients. 2021;14(1):23. doi:10.3390/nu14010023 35010898 PMC8747015

[58] National Health and Medical Research Council . *Australian dietary guidelines: providing the scientific evidence for healthier Australian diets/National Health and Medical Research Council*. National Health and Medical Research Council. 2013. Accessed March 1 2023. https://www.eatforhealth.gov.au/guidelines/guidelines

[59] Blanton CA , Moshfegh AJ , Baer DJ , Kretsch MJ . The USDA automated multiple‐pass method accurately estimates group total energy and nutrient intake. J Nutr. 2006;136(10):2594‐2599. 16988132 10.1093/jn/136.10.2594

